# Eight weeks of resistance exercise improves mood state and intestinal permeability in healthy adults: A randomized controlled trial

**DOI:** 10.14814/phy2.70219

**Published:** 2025-02-09

**Authors:** Emily Dow, Mario I. Hernandez, Carol S. Johnston

**Affiliations:** ^1^ College of Health Solutions Arizona State University Phoenix Arizona USA

**Keywords:** depression, inflammation, intestinal permeability, mental health, resistance training, strength training

## Abstract

To explore a potential link between resistance exercise and the gut‐brain axis, this study examined the impact of resistance exercise on intestinal permeability, as indicated by lipopolysaccharide binding protein (LBP), and mood state in healthy adults. Sedentary participants (*n* = 20; 39.5 ± 12.1 y; 27.4 ± 5.3 kg/m^2^) were randomly assigned to the resistance exercise (REX) or wait‐listed control (CON) groups. REX participants strength trained 3× weekly (advancing from 45%–55% to 70%–80% 1RM for 3–4 sets over 8 weeks). Strength testing, evaluation of mood states, and collection of fasting blood occurred at baseline and weeks 4 and 8. At baseline, LBP concentrations were inversely correlated to all strength measures (*r* range: −0.48 to −0.57; *p* < 0.05). The gain in total strength [(split squat left + right)/2 + bench press] was 45% higher for REX versus CON participants (*p* = 0.019), and serum LBP concentrations fell 16% for REX participants and rose 9% in CON participants (*p* = 0.014). Mood was significantly improved by resistance training versus control (but this improvement was not related to changes in LBP; *r* = −0.001). These findings support a role for resistance exercise in improving mood state and intestinal barrier function, but more research is warranted to further explore the effects of resistance training on the gut‐brain axis.

## 1 INTRODUCTION

A growing body of evidence supports a link between the gut microbiome and depressive disorders and mental well‐being, termed the “gut‐brain axis” (Tan et al., 2024). The intestinal barrier, a selectively permeable, multi‐layer surface separating the microbiota and host, plays a key role in this cross‐talk (Di Tommaso et al., 2021). Stressful situations can activate inflammatory conditions in the gut that adversely impact the microbiota secretory products and the permeability of the intestinal barrier (Marano et al., 2023). Tight junctions are intercellular adhesion complexes in the epithelia that control the transport of substances across the intestinal mucosa, and inflammation also disrupts these complexes, permitting excessive translocation of bacterial endotoxins such as lipopolysaccharide (LPS) into the systemic circulation. LPS heightens immune dysregulation and inflammatory cytokine release in many tissues, contributing to chronic conditions, including neuropathology and depressive symptomology (Di Tommaso et al., 2021; Gong et al., 2023; Li et al., 2017).

There is great interest in identifying effective interventions to improve intestinal barrier integrity to mitigate depressive disorders and mood disturbances. Resistance exercise is broadly recommended as it leads to gains in strength, lean body mass, and bone density, but resistance exercise also has positive effects on depression symptomology, particularly in individuals with diagnosed depression (Gordon et al., 2018; Rossi et al., 2024). Although mechanisms for these favorable effects on the brain have not been delineated, resistance exercise can attenuate intestinal injury and reduce systemic inflammation (Broadhouse et al., 2020; Jin et al., 2017), actions that could improve brain health via the gut‐brain axis. Previous studies investigating the effects of various exercise modalities on intestinal permeability have reported that acute exercise can induce intestinal barrier dysfunction and endotoxemia. These effects have been observed following both aerobic and resistance exercise (van Wijck et al., 2011, 2013). However, favorable chronic adaptations at the intestinal barrier level have primarily been associated with interventions incorporating aerobic exercise, highlighting the need for further research into resistance training‐specific adaptations. Recently, Moore et al. (Moore et al., 2022) conducted a secondary data analysis using metabolomics to examine changes in functional genes in stool samples from older male adults who completed a 6‐week supervised resistance training intervention. Pathway analyses indicated significant changes (pre to post) for gene activity linked to decreased lipopolysaccharide production and increased mucin biosynthesis, changes which are consistent with improved gut health. However, this secondary analysis lacked a control group. A randomized controlled investigation to directly examine the impact of resistance exercise on the intestinal barrier function would help elucidate links between resistance exercise and the gut‐brain axis.

The purpose of this study was to explore the impact of 8 weeks of resistance exercise training on relationships between intestinal permeability, systemic inflammation, and mood symptomology in healthy adult subjects. Based on the current body of literature, we hypothesized that an 8‐week resistance training intervention would lead to reductions in biomarkers of intestinal permeability and inflammation, as well as improvements in self‐reported mood states, in healthy adult participants. LPS is transported in blood bound to LPS‐binding protein (LBP), and serum LBP was measured in this study as a surrogate of LPS concentrations and intestinal permeability (Mohr et al., 2022). As described by Mohr et al., LBP is the preferred systemic biomarker for LPS given its high correlation with LPS levels, particularly at lower concentrations such as those expected to be observed among healthy individuals (Citronberg et al., 2016; Mohr et al., 2022; Moreno‐Navarrete et al., 2012; Schumann, 2011). In addition, serum concentrations of pro‐inflammatory cytokines were measured, and validated surveys of depression and mood states were utilized to quantify changes in emotional health.

## 2 MATERIALS AND METHODS

### 2.1 Study design

**FIGURE 1:**
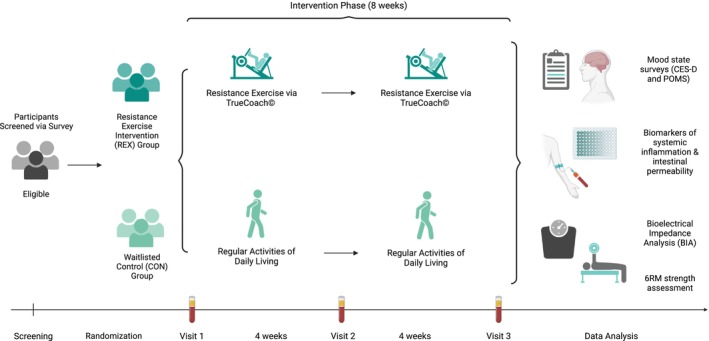
Timeline of study procedures (illustration created with BioRender.com).

A randomized, waitlisted controlled study design was employed. Participants were randomly allocated to either a resistance exercise intervention group (REX) or a waitlisted control group (CON). CON participants received the supervised exercise program following the completion of the study. Given the remote administration of the exercise program, REX participants were monitored closely by a certified personal trainer (National Strength and Conditioning Association; NCSA) via video feedback and direct messaging. Serum LBP and pro‐inflammatory cytokines were measured, and validated surveys of depression and mood states were utilized to quantify changes in emotional health. Strength was measured using submaximal 6 repetition maximum (6RM) testing for the bench press and split squat (American College of Sports Medicine, 2014), and body composition was measured using bioelectrical impedance (TANITA TBF‐400 NTEP Body Composition Analyzer; TANITA Corp, Arlington Heights, IL). Figure 1 displays the chronological flow for the study. It is important to note that although measurements were collected at three time points (weeks 0, 4, and 8), only pre‐ and post‐intervention data were analyzed to assess 8‐week changes.

### 2.2 Subjects

Participants were healthy men and women, 18–60 years of age, free of active medical conditions (including but not limited to heart disease, diabetes, prediabetes, stroke, cancer, asthma, gastrointestinal disorders, or psychiatric conditions), not taking any prescription drugs for managing chronic conditions, and able to participate in moderate to vigorous exercise as determined by the Physical Activity Readiness Questionnaire (PARQ) (American College of Sports Medicine, 2014). They were sedentary (e.g., scored <24 on the Godin‐Shepard Leisure Time Physical Activity Questionnaire [GSLTQ]) (Amireault & Godin, 2015); not actively strength training; able to access a full gym; and available for on‐site testing. Exclusion criteria included antibiotic use in the past 6 months; prebiotic, probiotic, or high‐dose antioxidant supplementation in the past 6 months; and adherence to a vegetarian diet. Before enrollment, participants agreed to maintain their typical sedentary behaviors, aside from study‐initiated exercise, and typical dietary patterns during the study. Once eligibility was verified, all participating individuals provided written informed consent. The study was approved by the Institutional Review Board at Arizona State University (STUDY00017316) and registered at clinicaltrials.gov (NCT05850221).

### 2.3 Sample size calculation and randomization

A sample size calculation was performed using G*Power with a minimum effect size of 0.68 and an alpha level of 0.05. The calculation determined that a total of 28 participants (*n* = 14 per group) were needed to achieve 80% power to detect improvements in 6‐repetition maximum (6RM) strength. This estimation was based on the results of a 5‐week training intervention described by Calatayud et al. (Calatayud et al., 2015). Participants were stratified based on age, sex, and BMI and randomized to the study groups by coin toss.

### 2.4 Procedures

#### 2.4.1 Resistance exercise intervention (REX) arm

The 8‐week exercise program was administered remotely via TrueCoach© (TC), an app platform designed for remote exercise, which allowed participants to complete training sessions in accordance with their schedules and at their desired gyms. REX participants had free access to the app for the length of the study. They were instructed to adhere to the assigned training program and avoid any additional moderate‐to‐intensive physical activity. Participants received instructions on how to properly interpret all program components (such as tempo, volume prescription, and rate of perceived exertion [RPE] scale) at baseline. This instructional time also served as an opportunity for participants to ask any preliminary questions and to receive instruction on how to perform all programmed exercises, which were selected for their relatively low‐skill, effective, and safe nature.

There were three training sessions weekly separated by a minimum of one complete rest day. Specific prescriptions for the number of sets, repetitions, and loading procedures were administered in the TC app, which included video demonstrations of each exercise. Training sessions lasted 45–60 min and included 5–7 resistance exercises performed at submaximal efforts (<90% 1RM), and a modified RPE scale ranging from 1 to 10 was used to approximate %1RM (Eston & Evans, 2009), as outlined by the American College of Sports Medicine (American College of Sports Medicine, 2014). The exercise intervention used progressive overload to improve strength and promote the accretion of lean body mass (Wernbom et al., 2007) by gradually increasing the intensity and number of sets (Baechle, 1989). Throughout the 8‐week intervention, the number of working sets increased from three to four, while repetitions per set decreased from 12 to 6. As a result, the prescribed intensities increased from 45%–55% of the estimated 1RM (corresponding to a modified RPE of 5–6 out of 10) to 70%–80% of the estimated 1RM (a modified RPE of 8–9 out of 10). Each training session targeted the following six movement patterns: squat/lunge, hinge, vertical press, horizontal press, vertical pull, and horizontal pull. To minimize differences in equipment access among REX participants, all exercises were performed using dumbbells and cables. During the first week, participants were encouraged to familiarize themselves with the equipment and determine appropriate weights for a challenging set of 12 repetitions, targeting a modified RPE of 8–9 out of 10. For each programmed training session, participants recorded the weights used for each exercise, the number of completed sets for each exercise, and uploaded at least one video per exercise to verify proper execution. The certified personal trainer communicated any necessary coaching points via the app, and exercise adherence was quantified for the REX participants as the total percentage of completed exercises throughout the 8‐week program as indicated on the TC app.

#### 2.4.2 Waitlisted control (CON) arm

CON participants were instructed to maintain their typical sedentary behavior throughout the 8‐week study period, and their activity was monitored via completion of the GSLTQ at each lab visit. Once the 8‐week study was completed, CON participants received the same exercise program as the REX group, and free access to the app, but were no longer study participants.

#### 2.4.3 Measurements

At study baseline and weeks 4 and 8, participants arrived at the testing facility following an overnight fast (no food or beverage with the exception of water for ≥10 h). Height was measured, and body weight, fat‐free mass (FFM; kg), body fat mass (BFM; kg), and BMI (kg/m^2^) were recorded utilizing the TANITA TBF‐400 NTEP Body Composition Analyzer (TANITA Corp, Arlington Heights, IL). Participants completed a multi‐pass 24‐h dietary recall. Dietary data was analyzed using Food Processor® Nutrition Analysis software (ESHA Research, Salem, OR) to determine energy intake (kcal) and the macronutrient composition, including carbohydrate (g), protein (g), fat (g), and fiber (g). Participants completed the Profile of Mood States (POMS) and the Center for Epidemiological Studies‐Depression (CES‐D) questionnaires. The POMS assessment utilizes an index of 65 adjectives describing six mood states (tension, anger, fatigue, depression, vigor, and confusion) over the prior 7 days (McNair et al., 1992). Participants were asked to respond to each adjective using a 5‐point Likert scale with responses ranging from “not at all” to “extremely”. Scores were computed for individual mood states as well as total mood state, with higher scores corresponding to greater mood disturbance. Scores >70 indicate possible clinical symptomology (Nyenhuis et al., 1999). The CES‐D contains 20 items that are rated on a four‐point scale based on how frequently over the prior week they experienced symptoms associated with depression (0—rarely or none of the time, 1—some or little of the time, 2—moderately or much of the time, or 3—most or almost all the time) (Radloff, 1977; Wallace & Milev, 2017). Scores range from 0 to 60, with scores >15 indicating an elevated risk of depression and scores >30 indicating elevated symptoms of depression that may influence the health of the individual (Wallace & Milev, 2017). The CES‐D has demonstrated strong reliability and validity in various at‐risk populations, including university‐aged adults experiencing subthreshold depression (Jiang et al., 2019).

Strength was assessed at each visit for the bench press and the split squat, thus quantifying both upper and lower body strength. For each exercise, participants began with one familiarization set of 12 repetitions before completing up to three 6‐repetition maximum (6RM) attempts with 3‐min rest intervals. Testing concluded once the participant was no longer able to perform more than 6 repetitions (American College of Sports Medicine, 2014). The order of exercise performance was standardized across participants, and, in the case of the split squat right and left (SSR and SSL), all subjects performed the test on their non‐dominant side followed by their dominant side. For both midpoint (week 4) and terminal (week 8) strength assessments, loads were adjusted based on previous 6RM scores coupled with an RPE scale ranging from 1 to 10 to account for strength gains acquired during the intervention. Both tests were performed using a collection of pre‐loaded and standard barbells ranging from 4.5 kg to over 90 kg, the loads of which could be increased by increments of 2.27 kg (5 lbs.) at a time.

#### 2.4.4 Serum biomarkers

Blood collected at weeks 0 and 8 was placed in serum separator tubes, held upright at room temperature for at least 30 min to clot, and centrifuged within 45 min of collection (2000 ***g*** for 10 min at 4°C). Serum aliquots were frozen (−80°C) for later LBP analysis via sandwich enzyme‐linked immunosorbent assay (ELISA) following the manufacturer's guidelines (Invitrogen Human LBP ELISA Kit; Catalog No. EH297RBX5). Serum cytokines were analyzed by human‐focused 15‐plex discovery assay (Human Cytokine Proinflammatory Focused 15‐Plex Discovery Assay® Array [HDF15]; Eve Technologies, Calgary, AB, Canada): anti‐inflammatory cytokines, IL‐1Ra, IL‐4, IL‐6, IL‐10, and IL‐13 (note that IL‐6 exerts an anti‐inflammatory effect in the context of exercise; Docherty et al., 2022); pro‐inflammatory cytokines, TNF‐α, IL1‐β, and IL‐8.

#### 2.4.5 Statistical analyses

Data are reported as the mean ± SD unless otherwise noted. Mann–Whitney *U* test was utilized to compare characteristics at baseline and the change data (week 8 values minus baseline values) between groups due to the small sample size. Spearman's rho test was performed to examine relationships between variables at baseline. Repeated measures ANOVA was used for POMS analyses to allow incorporation of the six sub‐scores into the calculation. All analyses were performed using IBM SPSS Statistics (IBM Corp. Released 2023. IBM SPSS Statistics for Windows, Version 29.0.2.0 Armonk, NY: IBM Corp). Significance was indicated at *p* values ≤0.05.

#### 2.4.6 Calculations

Whole‐body strength was calculated as the total load (kg) completed for the 6RM bench press and split squat combined. The load for the split squat was represented by the mean value between left and right sides, accounting for any variability between dominant and non‐dominant orientations. CES‐D was the sum of 20 items, four of which were reverse‐scored (Radloff, 1977). The total mood disturbance score for the POMS assessment was calculated as the sum of all 6 subdomains (tension, anger, fatigue, depression, vigor, and confusion) with vigor contributing a negative value, consistent with standard POMS scoring guidelines (McNair et al., 1992).

## 3 RESULTS

Study recruitment started in April 2023 and ended in July 2023. Of the 174 respondents to the online screener, 71 respondents qualified for the trial, and 23 respondents agreed to enroll in the study and provided written consent. Three participants were lost to attrition (1 REX and 2 CON), and data were analyzed for the 20 participants completing the study in its entirety (*n* = 11 and 9 for REX and CON respectively). There were no differences between groups at baseline for the demographic or outcome variables (Table 1). Protocol adherence (participation in three training sessions/week for 8 weeks) for REX participants ranged from 55% to 100% (mean: 85 ± 16%). Two individuals completed less than two‐thirds of the exercise sessions due to severe allergies and time constraints and were removed from the remaining analyses, raising protocol adherence to 91 ± 10% for the final nine REX participants. LBP concentrations were inversely related to SSL, SSR, and total strength at baseline (correlation coefficients ranged from −0.53 to −0.57, *p* < 0.05). LBP concentrations were not correlated to depression scores at baseline.

**TABLE 1:** Participant characteristics at baseline by group: REX (resistance exercise) and CON (wait‐listed control)^a^.

	REX	CON	*p*
M/F	1/10	2/7	0.850
Age, y	39.6 ± 10.0	39.3 ± 14.8	0.882
Body weight, kg	76.5 ± 19.2	78.1 ± 15.3	0.824
BMI, kg/m^2^	27.1 ± 5.0	27.8 ± 5.8	0.824
Body fat, %	33.4 ± 9.2	33.9 ± 11.0	1.000
FFM, kg	49.7 ± 13.5	50.4 ± 6.5	0.456
Total strength, kg	576 ± 387	653 ± 318	0.295
GSLTQ score	12.5 ± 9.3	16.4 ± 7.1	0.456
CES‐D score	7.5 ± 9.5	11.0 ± 7.8	0.175
Total POMS score	16.3 ± 33.5	18.1 ± 23.1	0.603
LBP, mg/mL	25.1 ± 7.3	20.2 ± 6.0	0.175

Abbreviations: BMI, Body Mass Index; CES‐D, Center for Epidemiological Studies‐Depression; F, female; FFM, Fat‐Free mass; GSLTQ, Godin‐Shepard Leisure Time Physical Activity Questionnaire; LBP, Lipopolysaccharide Binding Protein *p* value represents Chi Square (sex) and Mann Whitney Tests; M, male; POMS, Profile of Mood States.

^a^ Data are mean ± SD. Total strength: split squats (R and L averaged) + bench press.

Changes from baseline in body weight, BMI, body fat percentage, and fat‐free mass did not differ between groups at study at week 8 (Table 2). Total strength [(6RM SSL + 6RM SSR)/2 + 6RM bench press] increased 74% and 39% in REX and CON participants, respectively, over the course of the 8‐week intervention, a difference between groups that achieved statistical significance (*p* = 0.019; Table 2). The average LBP concentration in serum fell 16% for REX participants during the intervention compared to a 9% increase in CON participants (*p* = 0.014; Table 3). Controlling for age or body composition did not impact the significance of the change in LBP or total strength between groups, and these variables were not related to LBP or total strength measures at baseline. However, the 8‐week change in total strength was not related to the 8‐week change in LBP (*r* = −0.214; *p* = 0.394) among the participants.

**TABLE 2:** Participant data at baseline and study weeks 4 and 8 (REX: Resistance exercise, *n* = 9; CON: Wait‐listed controls, *n* = 9). Change data (∆) are week 8 data minus baseline data^a^.

		Baseline	Week 4	Week 8	∆	*p*
Body weight, kg	REX	80.4 ± 16.4	78.8 ± 14.9	81.8 ± 170	1.4 ± 1.7	
	CON	78.1 ± 15.3	78.3 ± 15.3	78.8 ± 14.4	0.7 ± 1.9	0.489
BMI, kg/m^2^	REX	27.5 ± 4.3	27.7 ± 4.2	27.9 ± 4.2	0.38 ± 0.58	
	CON	27.8 ± 5.8	27.7 ± 5.6	27.9 ± 5.4	0.18 ± 0.76	0.796
Body fat, %	REX	34.9 ± 6.8	35.0 ± 6.8	34.5 ± 6.7	−0.33 ± 0.87	
	CON	33.3 ± 10.9	33.3 ± 10.9	33.8 ± 10.7	−0.16 ± 1.74	0.863
FFM, kg	REX	51.7 ± 13.8	51.1 ± 10.6	53.8 ± 14.1	2.0 ± 2.7	
	CON	50.4 ± 6.5	51.1 ± 7.2	51.1 ± 6.5	0.7 ± 1.2	0.297
Total strength^b^, 6RM [kg]	REX	48.02 ± 30.83	64.87 ± 31.57	83.51 ± 46.12	35.49 ± 22.80	
	CON	49.44 ± 24.08	58.78 ± 28.39	68.95 ± 31.74	19.51 ± 24.51	**0.019**
CES‐D score	REX	4.44 ± 3.84	5.22 ± 2.44	3.44 ± 2.24	−1.00 ± 4.69	
	CON	11.00 ± 7.79	13.33 ± 7.87	15.00 ± 12.76	4.00 ± 10.15	0.222
POMS score^c^	REX	6.4 ± 13.9	5.0 ± 23.7	−2.0 ± 13.6	−8.4 ± 19.8	
	CON	18.1 ± 23.1	23.7 ± 22.6	25.8 ± 30.3	7.7 ± 16.1	**0.018**

Abbreviations: 6RM, 6‐Repetition Maximum; BMI, Body Mass Index; CES‐D, Center for Epidemiological Studies‐Depression; FFM, Fat‐Free Mass; POMS, Profile of Mood States.

^a^ Data are mean ± SD; two REX participants removed as weight training schedule adherence was <65%. *p* value represents Mann Whitney test for change data.

^b^ Total strength: Split Squat 6RM + Bench Press 6RM. Split Squat 6RM: (Split Squat 6RM (right) + Split Squat 6RM (left))/2.

^c^ Analysis incorporated the six sub‐scores utilizing repeated Measures ANOVA.

**TABLE 3:** LBP and cytokine data at baseline and study week 8 (REX: Resistance exercise, *n* = 9; CON: Wait‐listed controls, *n* = 9). Change data (∆) are week 8 data minus baseline data^a^.

		Baseline	Week 8	∆	*p*
LBP, mg/L	REX	26.3 ± 7.3	22.0 ± 5.4	−4.3 ± 7.0	
	CON	20.2 ± 6.0	22.1 ± 4.5	1.9 ± 5.1	**0.014**
Anti‐inflammatory cytokines					
IL‐1Ra, pg/mL	REX	8.89 ± 6.06	7.95 ± 5.02	−0.94 ± 3.19	
	CON	9.56 ± 8.50	5.84 ± 4.08	−3.72 ± 6.08	0.161
IL‐4, pg/mL	REX	1.37 ± 1.72	1.82 ± 3.03	0.45 ± 1.38	
	CON	0.55 ± 0.31	0.42 ± 0.27	−0.13 ± 0.12	0.077
IL‐6, pg/mL	REX	4.47 ± 5.56	3.91 ± 6.71	−0.51 ± 3.41	
	CON	2.64 ± 3.00	1.67 ± 1.80	−0.97 ± 1.57	0.796
IL‐10, pg/mL	REX	2.99 ± 1.48	2.74 ± 1.59	−0.25 ± 1.10	
	CON	2.83 ± 1.37	2.48 ± 1.44	−0.34 ± 0.53	0.863
IL‐13, pg/mL	REX	83.3 ± 110.1	96.6 ± 138.9	13.3 ± 38.5	
	CON	51.4 ± 52.2	38.4 ± 34.9	−13.0 ± 23.9	0.222
Inflammatory cytokines					
TNF‐ɑ, pg/mL	REX	37.6 ± 15.9	40.6 ± 17.3	2.95 ± 7.13	
	CON	33.8 ± 15.2	31.6 ± 15.0	−2.18 ± 3.48	0.077
IL‐1β, pg/mL^b^	REX	4.40 ± 6.87	6.46 ± 14.6	2.06 ± 7.96	
	CON	3.59 ± 4.34	2.31 ± 3.37	−1.27 ± 1.75	0.239
IL‐8, pg/mL	REX	9.86 ± 7.09	9.98 ± 3.72	0.113 ± 4.08	
	CON	9.42 ± 7.15	8.65 ± 5.06	−0.77 ± 2.80	0.222

^a^ Data are mean ± SD; two REX participants removed as weight training schedule adherence was <65%. *p* value represents Mann Whitney test for change data.

^b^ One outlier removed from REX group (>3 SD from mean).

**FIGURE 2:**
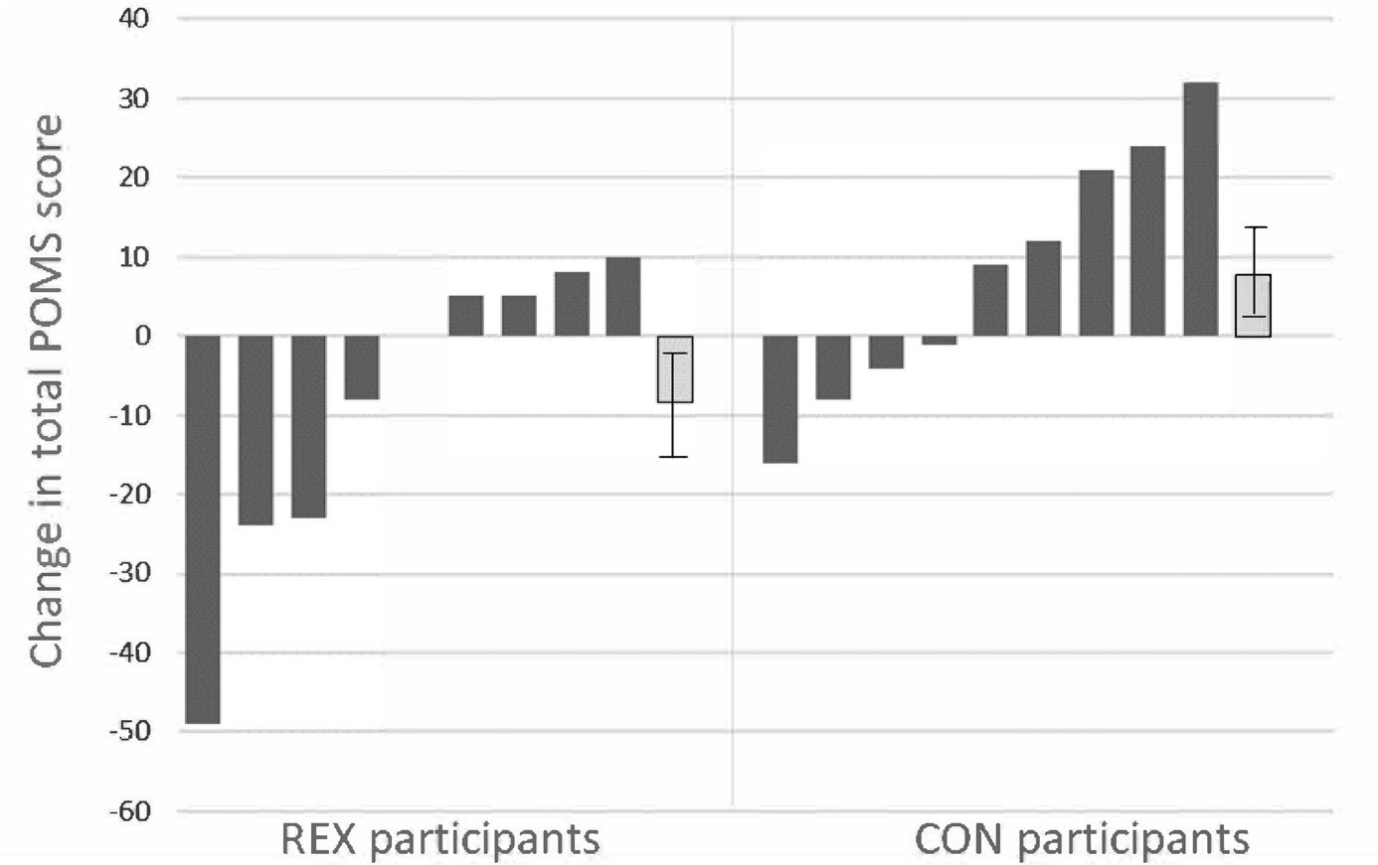
Dark bars represent Individual participant data for the change in total POMS scores by group: REX (resistance exercise, *n* = 9) and CON (wait‐listed controls, *n* = 9). Change data are week 8 data minus baseline data. Light bars represent the mean ± SEM for group data (REX, −8.4 ± 6.6; CON, +7.7 ± 5.4; *p* = 0.018).

Although CES‐D scores fell 23% in REX participants and rose 36% in CON participants over the course of the study, this difference between groups was not significant (*p* = 0.222; Table 2). However, the 8‐week change in mood states as indicated by the POMS scores (decreasing in REX participants and increasing in CON participants) did differ significantly between groups *p* = 0.018; Table 2, and post‐hoc analyses demonstrated that the vigor/activity score improved significantly, and the confusion/bewilderment score trended towards improvement, in REX participants in comparison to the CON participants (*p* = 0.014 and *p* = 0.10, respectively; Figure 2). The 8‐week change in LBP concentrations was not related to the 8‐week change in POMS scores (*r* = −0.001; *p* = 0.997) among the participants. The 8‐week changes in the anti‐inflammatory cytokines (IL‐1Ra, IL‐4, IL‐6, IL‐10, and Il‐13) and the pro‐inflammatory cytokines (TNF‐ɑ, IL‐1β, and IL‐8) did not differ between the study arms (Table 2). Mean daily energy intake and dietary carbohydrate, protein, fat, and fiber did not differ between study groups at baseline and showed no significant changes over the 8‐week study period (data not shown).

## 4 DISCUSSION

This study evaluated the impact of an 8‐week resistance exercise intervention on intestinal permeability and mood state in healthy adults. Unlike a majority of trials that examined the impact of exercise in adults with depressive symptoms (see Gordon et al., 2018), healthy adults were recruited for this study since depression in adults is on the rise in the U.S., and strategies to improve mental well‐being and reduce risk for developing depressive symptoms are needed (Gordon et al., 2018). At baseline, all strength measures were inversely correlated to LBP concentrations in the study participants. At the end of the 8‐week study, resistance exercise increased total strength (+74% and +39% for REX and CON, respectively; *p* = 0.019) and favorably impacted LBP concentrations in REX vs. CON (−16% and +9%, respectively; *p* = 0.041). The gain in strength was consistent with previous resistance exercise trials of similar duration in untrained participants (Campos et al., 2002; Schoenfeld et al., 2021). Mood state was also improved in REX participants in comparison to CON participants; however, the changes in mood state, LBP concentrations, and total strength were not correlated. Hence, the exercise‐stimulated improvement in gut barrier function (e.g., the lowering of LBP) did not appear to be associated with the observed gains in muscle strength or mood state.

Data investigating the efficacy of remote resistance exercise interventions is still in its infancy; however, growing interest has been identified, particularly in a post‐pandemic landscape, where previous reports have indicated that up to 89% of regular exercisers transitioned to virtual personal training during lockdowns (Carlson et al., 2022). Many interventions have focused on home‐based exercise programs using minimal equipment, such as bodyweight or resistance bands. Remote exercise monitoring has generally been shown to be safe and achieves adherence rates comparable to those of in‐person training interventions (Carlson et al., 2022; Ferrari et al., 2024). Similarly, remote exercise programming has demonstrated comparable effects on body composition and strength, while maintaining key aspects of traditionally supervised training, including intensity of effort, motivation, enjoyment, and supervision quality (Carlson et al., 2022; Ferrari et al., 2024). These interventions often include real‐time remote supervision via video platforms and/or biofeedback monitoring through wearable devices, presenting an opportunity to combine the convenience of remote administration with the empirical benefits of modern technology. However, other investigators have noted potential trade‐offs, such as prioritizing low‐skill, safer exercises at the expense of potentially more effective alternatives (Ferrari et al., 2024). The current study demonstrates the potential of highly accessible, gym‐based remote programming to effectively improve strength, mood, and gut barrier integrity, with high adherence and no reported adverse events.

Data linking resistance training to intestinal barrier function are scarce. Santana‐Oliveira et al. recently demonstrated in mice with dysbiosis, an imbalanced pathological gut microbiome, that high‐intensity interval training (HIIT; an exercise protocol that resembles resistance training) preserved the expression of the tight junction proteins that maintain an effective intestinal barrier (Santana‐Oliveira et al., 2023). The tight junction protein, occludin, was enhanced 753% in the HIIT mice compared to control. Concomitantly, plasma lipopolysaccharide concentrations and LBP expression in brown adipose tissue were reduced 7% and 68%, respectively, in the HIIT mice and increased in the control mice (Santana‐Oliveira et al., 2023). In obese Zucker rats, HIIT upregulated occludin over 100%, and plasma LBP was reduced, relative to untrained rats (Maillard et al., 2019). In humans, a single report suggested that resistance training increased gene activity linked to increased mucin biosynthesis; however, this was a secondary analysis that lacked a control group (Moore et al., 2022). Mucins are a key component of a healthy gut mucosa. These glycoproteins are hydrated following release from goblet cells and expand to form a gel that blankets the epithelia to block the passage of toxins and bacteria (Capaldo et al., 2017).

Although little data is available concerning the chronic effects of resistance training on intestinal barrier permeability and circulating cytokine profiles, there is a mounting body of evidence that suggests aerobic training improves gut barrier integrity across a range of exercise modalities. Cross‐sectional research suggests that trained cyclists present with threefold lower levels of plasma LPS at rest compared to sedentary adults with comparable body mass indices (Lira et al., 2010). In 2020, significant reductions in serum LBP were observed by Motiani et al. in response to 2 weeks of moderate aerobic exercise among individuals with insulin resistance (Motiani et al., 2020). These findings are further supported by those of Pasini et al., which demonstrated that 6 months of combined exercise (endurance, resistance, and flexibility training) produced significant reductions in zonulin, a protein understood to destabilize tight junctions (Pasini et al., 2019). Further, a 2021 trial by Feng et al. suggested that 12 weeks of combined aerobic and resistance exercise may induce favorable improvements in fitness capacity, quantified by VO2 max, which were inversely related to serum I‐FABP, a marker for intestinal epithelial damage (Feng et al., 2021). Although the mechanisms underlying improvements in intestinal barrier function remain unclear, insights from exercise‐induced intestinal hyperpermeability may provide a plausible explanation (van Wijck et al., 2011, 2013). Similar to the acute pro‐inflammatory response elicited by both aerobic and resistance exercise, which subsequently drives anti‐inflammatory adaptations, the transient stress placed on the intestinal barrier during exercise may trigger adaptations that enhance its resilience over time (Chow et al., 2022; Zunner et al., 2022). While further investigation is needed, this hypothesis could explain the chronic reductions in LBP observed in the present study. The findings of the present study, indicating a significant reduction in circulating LBP among REX participants, contribute novel insights linking resistance training specifically to reduced intestinal permeability.

Improvements in intestinal integrity are generally linked to reductions in the inflammatory milieu in the intestinal lumen. Immune system activation, as indicated by increases in inflammatory cytokines, has been linked to increased intestinal permeability (Al‐Sadi et al., 2008, 2010; Barreau et al., 2004). In vitro, IL‐1β‐activated mitogen‐activated protein kinases stimulate NF‐κB activity and the opening of intestinal epithelial tight junctions (Al‐Sadi et al., 2008, 2010). Conversely, probiotics enhance gut microbiota fermentation and the production of short‐chain fatty acids that reduce inflammation in the gut, which improves tight junction barrier function (Shinde et al., 2019). Additionally, there is evidence that probiotics may also improve tight junction barrier function by suppressing the NF‐κB inflammatory pathway (Chen et al., 2021; Good et al., 2014). In the present report, strength acquisition was linked to significant reductions in LBP, suggesting improved intestinal integrity, but did not impact circulating cytokine concentration. It is postulated that the changes in cytokine profiles in response to regular exercise may be due, in part, to reductions in body fat mass since adipocytes are a primary source of pro‐inflammatory cytokines such as TNF‐α (Winn et al., 2021). Body fat percentage did not improve significantly in the REX participants in comparison to control. However, reductions in pro‐inflammatory cytokines in response to exercise are also observed in the absence of body composition changes and/or weight loss (Shinde et al., 2019; Winn et al., 2021). More research is warranted to corroborate the beneficial impact of resistance training specifically on intestinal integrity and to delineate the mechanisms involved.

POMS scoring improved significantly for REX participants vs. CON participants (−131% and +43%, respectively), and although CES‐D scores did not differ significantly between groups over the course of the study, scores did move in similar directions as the POMS scores (−23% and +36%, respectively, for the REX and CON participants). These reductions in scores likely have physiological relevance since the commonly agreed definitions for “clinical symptom response” in the psychology field are a treatment response at 50% change or a partial treatment response at 25% change on a valid symptom rating scale (Lecrubier, 2002; Wise, 2004). However, it is important to note that participants scored well below the cutoffs that indicated emotional symptomology on both the POMS and CES‐D measures; thus, further improvements in these scores would be less probable. Although CES‐D and POMS scores appeared different between the REX and CON groups at baseline, these differences were not statistically significant. The change data (week 8 values minus baseline values) was significantly different between groups with mood improving in the REX versus CON groups. Controlling for baseline POMS scores did not erase this significance between groups. There is a lack of studies specifically focused on resistance training and mood state, but studies in elderly adults utilizing elastic band resistance training demonstrated improvements mental health including changes in total POMS scores, similar to those noted herein (Winn et al., 2021).

As with the observed improvements in intestinal barrier function, the mechanisms underlying enhancements in mood state remain unclear and are likely multifactorial. Prior research suggests that mood improvements and reductions in depressive symptoms may stem from physiological factors, including increased production of neurotransmitters such as serotonin, endorphin release, elevated brain‐derived neurotrophic factor (BDNF), regulation of the hypothalamic–pituitary–adrenal (HPA) axis, reduced inflammation, and improved sleep quality (Alizadeh Pahlavani, 2024; Sun et al., 2023). Psychosocial factors, such as enhanced self‐efficacy, a greater sense of community, and distraction from negative thoughts, have also been implicated (Bahrke & Morgan, 1978; Chen et al., 2015; Deforche & De Bourdeaudhuij, 2015; Eyre et al., 2013).

This study explored the hypothesis that structural improvements in the intestinal barrier could reduce the translocation of harmful substances, such as endotoxin (measured via LBP), into circulation. Endotoxin has the potential to cross the blood–brain barrier, triggering a pro‐inflammatory response that may contribute to depressive symptoms. Elevated endotoxemia has indeed been linked to depressive symptomology in both baseline conditions and experimentally induced states, even in the absence of gastrointestinal symptoms (De La Garza, 2005; Lasselin et al., 2021; Madison et al., 2020). Although the exploration of this mechanism is in its early stages, our findings align with the idea that enhanced intestinal barrier integrity may coincide with improvements in mood and depressive symptoms. However, these improvements were not significantly associated in the present study, warranting further investigation to clarify any potential relationships.

The final sample size (*n* = 20) fell short of the initially calculated requirement (*n* = 28), representing a limitation of the present study despite the achievement of significant findings. Recruitment and retention challenges are well‐documented in human exercise interventions, particularly for time‐intensive protocols such as this 3‐day‐per‐week, 8‐week program. Similar studies typically report sample sizes ranging from 23 to 25 participants, with attrition rates between 20% and 50% (Linke et al., 2011; Swinton et al., 2024; Yagiz et al., 2022). Among those who complete such studies, adherence rates average approximately 66%. The final sample size in this study reflects these broader trends, accounting for participants lost to attrition and those with low adherence (completion of less than two‐thirds of the intervention). Notably, adherence among participants who remained and met completion criteria rose to 91 ± 10%, suggesting a potential benefit of remote exercise programming due to its convenience and flexibility.

REX participants may have engaged in exercise within 24 h of blood sampling, and this is an important study limitation. Since exercise impacts acute and resting cytokine profiles differently (e.g., inflammatory cytokines are raised acutely but reduced when measured at rest; Docherty et al., 2022), the timing of the assessment should be clearly defined (Pedersen & Hoffman‐Goetz, 2000). In the present study, participants were not instructed to avoid exercise for any specified length of time prior to serum collection, so sampling may have taken place between 12‐ and 24‐h post‐exercise. It is noteworthy that the CON group experienced a cumulative 39% improvement in functional strength assessments, albeit a change that was significantly less than that observed for REX participants (+74%). This finding underscores the importance of employing a control group when assessing functional strength outcomes, particularly among individuals with limited experience with resistance exercise. The practice effects influencing such assessments are well‐documented, and it is recommended that in the case of inexperienced trainees, two to three test sessions may be necessary to accurately assess maximal strength (Winn et al., 2021). Given that only one baseline assessment was conducted in the present study, the observed strength improvements both in the REX and CON groups may be partially attributed to practice effects. Although the study team used consistent language in their instructions for all 6RM testing—with the same team member conducting all assessments—complete novices may respond differently to these instructions compared to after they have completed the assessment multiple times. Neural adaptations are known to contribute to early strength improvements in response to resistance exercise (Gabriel et al., 2006; Sale, 1988). Therefore, gaining experience with the tested movement patterns may result in apparent strength gains without muscle hypertrophy. This is particularly relevant for unilateral exercises like the split squat, which have high stability demands.

Another limitation of the present study was the inability to detect potential sex differences in response to the intervention due to our limited sample size and the unequal distribution of male (*n* = 3) and female (*n* = 15) participants. Efforts were made to mitigate sex‐related variability by balancing participants between study groups and accounting for hormonal fluctuations through measurements taken at 4‐week intervals. While the underrepresentation of male participants is a limitation, the focus on female subjects addresses a significant gap in exercise science literature, which often underemphasizes this population (Cowley et al., 2021; Costello et al., 2014; James et al., 2023; Smith et al., 2022). Nonetheless, further research is needed to explore sex‐specific responses to resistance exercise, particularly concerning mood state and intestinal permeability, given the mixed findings previously reported for strength and hypertrophy adaptations (Roberts et al., 2020). Other study limitations include the small sample size, the lack of direct exercise supervision, and reliance on self‐reported behaviors and assessments.

## 5 CONCLUSION

To our knowledge, this is the first trial to examine directly the impact of resistance training on the integrity of the intestinal barrier and its associations with depressive disorders and mood states in humans utilizing a randomized controlled study design. The data demonstrate that the 8‐week resistance exercise intervention significantly enhanced total strength, reduced serum LBP, and improved mood states, although the improvement in strength and emotion was not related to improvements in intestinal barrier function. These findings contribute novel insights into the benefits of resistance exercise, extending beyond physical strength gains, to encompass potential improvements in gut health and mental well‐being. Future research is warranted to further elucidate the mechanisms underlying these benefits and to explore the long‐term effects of resistance training on intestinal permeability and psychological health.

## AUTHOR CONTRIBUTIONS

Research Conception/Design: ED, CSJ; Data Collection: ED, MIH; Data Analysis: ED, CSJ; Interpretation: ED, CSJ; Figure Preparation: ED, CSJ; Manuscript Preparation: ED, CSJ; Manuscript Approval: ED, MIH, CSJ.

## FUNDING INFORMATION

No external financial support or supplies were provided during this study. Internal funding was provided by the Graduate and Professional Student Association at Arizona State University.

## CONFLICT OF INTEREST STATEMENT

The authors have nothing to report.

## ETHICS STATEMENT

All experimental protocols were approved by the Institutional Review Board at Arizona State University (STUDY00017316) and registered at clinicaltrials.gov(NCT05850221). All methods were carried out in accordance with relevant guidelines and regulations. Informed consent was obtained from all participants before inclusion in the study.

## DISCLAIMERS

The authors have no disclaimers to report.

## Data Availability

The data that support the findings of this study are available from the corresponding author upon reasonable request.
